# Rationale for revision and proposed changes of the WHO diagnostic criteria for polycythemia vera, essential thrombocythemia and primary myelofibrosis

**DOI:** 10.1038/bcj.2015.64

**Published:** 2015-08-14

**Authors:** T Barbui, J Thiele, A M Vannucchi, A Tefferi

**Affiliations:** 1 Hematology and Research Foundation, Papa Giovanni XXIII Hospital, Bergamo, Italy; 2 Institute of Pathology, University of Cologne, Cologne, Germany; 3 Department of Experimental and Clinical Medicine, Azienda Ospedaliera-Universitaria Careggi, University of Florence, Florence, Italy; 4 Hematology Division, Mayo Clinic, Rochester, MN, USA

## Abstract

The 2001/2008 World Health Organization (WHO)-based diagnostic criteria for polycythemia vera (PV), essential thrombocythemia (ET) and primary myelofibrosis (PMF) were recently revised to accomodate new information on disease-specific mutations and underscore distinguishing morphologic features. In this context, it seems to be reasonable to compare first major diagnostic criteria of the former WHO classifications for myeloproliferative neoplasm (MPN) and then to focus on details that have been discussed and will be proposed for the upcoming revision of diagnostic guidelines. In PV, a characteristic bone marrow (BM) morphology was added as one of three major diagnostic criteria, which allowed lowering of the hemoglobin/hematocrit threshold for diagnosis, which is another major criterion, to 16.5 g/dl/49% in men and 16 g/dl/48% in women. The presence of a *JAK2* mutation remains the third major diagnostic criterion in PV. Subnormal serum erythropoietin level is now the only minor criterion in PV and is used to capture *JAK2*-unmutated cases. In ET and PMF, mutations that are considered to confirm clonality and specific diagnosis now include *CALR*, in addition to *JAK2* and *MPL*. Also in the 2015 discussed revision, overtly fibrotic PMF is clearly distinguished from early/prefibrotic PMF and each PMF variant now includes a separate list of diagnostic criteria. The main rationale for these changes was to enhance the distinction between so-called masked PV and *JAK2*-mutated ET and between ET and prefibrotic early PMF. The proposed changes also underscore the complementary role, as well as limitations of mutation analysis in morphologic diagnosis. On the other hand, discovery of new biological markers may probably be expected in the future to enhance discrimination of the different MPN subtypes in accordance with the histological BM patterns and corresponding clinical features.

Following the 2001^(ref. 1)^ and 2007/2008^(refs. 2, 3, 4)^ updated classifications of myeloproliferative neoplasms (MPNs) by the World Health Organization (WHO) a number of clinical-pathological studies have documented the usefulness of these relevant diagnostic criteria.^5, 6, 7, 8, 9, 10, 11^ However, lively discussion and occasionally controversy persist as concerns not only the reliability of the proposed clinical features required for WHO-defined diagnosis, but particularly the diagnostic relevance as well as the reproducibility of bone marrow (BM) morphology criteria.^12, 13, 14, 15, 16^

Moreover, recently evolving molecular features yielded new perspectives and provided novel insights for the understanding 'of the pathobiology of MPN. In this context, the discovery of calreticulin (*CARL*) mutations occurring in *JAK2*- and *MPL*-negative essential thrombocythemia (ET) and primary myelofibrosis (PMF)^17, 18^ facilitated diagnostic precision and may also harbor prognostic relevance.^19^

Overall, in consideration of the new molecular findings and experiences gained in the past years, there is a rationale to make proposals for the upcoming revision of diagnostic guidelines that are going to be published in the near future.

The set of the reasons on which a WHO revision is suggested are the following:

1. The description of novel molecular findings other than Janus kinase (*JAK2*V617F) and myeloproliferative leukemia virus oncogene (*MPL*) mutations that deserve diagnostic importance for all the three major entities.
2. In polycythemia vera (PV), the possibility of an underdiagnosis as the consequence of applying the former hemoglobin (Hb) or hematocrit (Hct) threshold values and the emerging role of BM morphology as a major criterion.
3. In ET, the need to differentiate ‘true ET' from prefibrotic/early primary PMF (prePMF) by emphasizing the lack of reticulin fibrosis at onset, owing that such distinction has prognostic implications.
4. In PMF, the challenge of defining more explicitly minor clinical criteria that may have major impact not only on accurate diagnosis but also on prognosis.

Altogether it seems to be reasonable to compare major diagnostic criteria of the former WHO classifications for MPN^1, 2, 3, 4^ starting from 2001, and then to focus on details suggested for the upcoming revision of diagnostic guidelines that, however, have not been published yet and therefore should be regarded as proposals.

## Mutations and their diagnostic and prognostic utility

From 2005 onwards, more than 20 somatic mutations have been described in MPN.^20^ The first mutation described (that is, *JAK2*V617F), which is also the most frequent, involves Janus kinase 2 (*JAK2* located on chromosome 9p24).^21^ *JAK2*V617F mutational frequency is estimated at 97% in PV, 50–60% in ET and 55–65% in PMF.^20^ However, *JAK2*V617F is not specific to MPN and is also seen in less than 5% of patients with acute myeloid leukemia, myelodysplastic syndromes (MDS), chronic myelomonocytic leukemia and other myeloid malignancies.^22^ Of note, *JAK2*V617F mutational frequency as high as 50% has been reported in the MDS/MPN overlap syndrome of refractory anemia with ring sideroblasts associated with marked thrombocytosis.^3^ Subsequent to the discovery of *JAK2*V617F, other *JAK2* mutations (for example, *JAK2* exon 12 mutations) were described in *JAK2*-unmutated PV and characterized by predominantly erythroid myelopoiesis.^23, 24^ *JAK2* mutations are not present in the normal control population and in patients with non-clonal erythrocytosis or thrombocytosis.^25^ Accordingly, *JAK2* mutation screening was adopted, as a useful clonal marker, by the WHO system of hematopoietic tumor classification, in its 2008 revised document.^3, 4^ In routine clinical practice, the absence of a *JAK2* mutation makes the diagnosis of PV unlikely but its presence cannot, by itself, distinguish PV from ET or PMF, thus the need for BM examination. Rare cases of *JAK2*-unmutated ‘PV' have been associated with *LNK*.^26^

The second most frequent mutations in MPN involve the *CALR-* gene located on chromosome 19p13.2.^18, 19^ With the exception of isolated reports,^27^ *CALR* mutations are absent in PV, whereas their frequencies are estimated at 20–25% in ET and PMF;^28^ therefore, *CALR* mutations appear to be relatively specific to ET and PMF. The third MPN-specific mutation involves the myeloproliferative leukemia virus oncogene (*MPL*; located on chromosome 1p34) and has been described in 3% of patients with ET and 7% of those with PMF, and only rarely in PV.^29, 30^ *JAK2*, *CALR* and *MPL* mutations are often mutually exclusive, although concurrent occurrence has been reported, likely as the result of concurrent individual mutated clones.^31^ Taken together, approximately 99% of PV patients are expected to harbor *JAK2* mutations, whereas 80–90% of patients with ET or PMF carry one of the three ‘MPN-driver' mutations: *JAK2*, *CALR* or *MPL*. It was therefore appropriate to suggest that the upcoming revision of the WHO system in addition to *JAK2* includes *CALR* and *MPL* mutation screening (Tables 1–4) in the diagnostic process for *JAK2*-unmutated ET and PMF.^28^ Once again, BM examination is often necessary for accurate diagnosis since *JAK2*/*CALR*/*MPL* mutation screening, by itself, cannot distinguish masked PV from *JAK2*-mutated ET, WHO-defined ET from prefibrotic/early PMF or triple-negative ET from other causes of thrombocytosis. The latter stems from the fact that about 10–15% of patients with ET or PMF lacks all three MPN driver mutations and are referred to as being ‘triple-negative'.^19^ In *JAK2*/*CALR*/*MPL*-mutated patients, mutant allele burden is sometimes helpful in further clarifying the specific diagnosis; for example, higher than 40% *JAK2* mutant allele burden is unusual in ET and suggests either masked PV or prefibrotic/early PMF.^32^

*JAK2*/*CALR*/*MPL* mutations also provide important prognostic information in MPN. A low *JAK2*V617F allele burden in PMF has been associated with worse prognosis,^33, 34^ whereas a higher than 50% *JAK2*V617F allele burden in PV has been associated with increased risk of fibrotic transformation.^35^ Also in PV, a higher *JAK2* mutant allele burden has been associated with pruritus.^36^ In ET and PMF, *JAK2* mutations are associated with older age, higher hemoglobin level, leukocytosis and lower platelet count and, in ET, an increased risk of thrombosis (compared to those with *CALR* mutation or triple-negative mutational status).^37^ Similarly, *CALR* mutations in ET and PMF have been associated with younger age and higher platelet count whereas their effect on Hb and leukocyte count appears to be different in ET (lower Hb and leukocyte count) versus PMF (higher Hb and leukocyte levels).^28^ Furthermore, among *CALR*-mutated ET patients, those with type 2 variant *CALR* mutation (a 5-bp TTGTC insertion; p.K385fs*47), compared to those with type 1 variant (a 52-bp deletion; p.L367fs*46) display higher platelet counts.^38^ More importantly, in PMF, the presence of type 1 or type 1-like *CALR* mutations has been associated with superior survival.^39^

Several other mutations in MPN are neither sensitive nor specific enough to warrant their consideration in the initial diagnostic process for MPN.^40^ The most frequent among these are *ASXL1* (Additional Sex Combs-Like 1), *TET2* (TET oncogene family member 2), *SRSF2* (serine/arginine-rich splicing factor 2) and *U2AF1* (U2 Small Nuclear RNA Auxiliary Factor 1) and the less frequent *IDH1*/*IDH2* (isocitrate dehydrogenase 1 and 2), *TP53* (tumor protein p53), *DNMT3A* (DNA cytosine methyltransferase 3a), *IKZF1* (IKAROS family zinc finger 1), *LNK* (*SH2B3*), *SF3B1* (splicing factor 3B subunit 1), *EZH2* (enhancer of zeste homolog 2), *CBL* (Casitas B-lineage lymphoma proto-oncogene) and *SETBP1* (SET binding protein 1).^40, 41, 42^ Interestingly, as reported also for *JAK2* some of these mutations (for example, *TET2*, *ASXL1* and *DNMT3A*) were recently shown to occur in ‘normal' elderly individuals (up to 5–6% in people older than 70 years).^43, 44, 45^ Regardless, in PMF, *ASXL1*, *SRSF2*, *EZH2*, *IDH1/2* and *U2AF1* mutations, as well as the number of such mutations in an individual patient, have been associated with shortened survival.^42, 46^ Finally, in terms of pathogenetic mechanisms, *JAK2* and *MPL* mutations are believed to drive myeloproliferation through direct activation of *JAK-STAT*, whereas megakaryocytes might be the primary target for *CALR* mutations that at least in part may still signal through the *JAK/STAT* pathway.^47^ Furthermore, most recently, the phenotype-patterning effect of concurrently occurring multiple mutations and their order of acquisition has been suggested.^47, 48, 49^

## Polycythemia vera

The key diagnostic issue in PV is which red cell parameters (Hb, Hct) may be more conveniently used as a surrogate of an increased red cell mass as measured by the isotope test.^12, 50, 51, 52, 53, 54^ According to the 2007/2008 updated WHO classification,^2, 3, 4^ the first major diagnostic criterion requires one of the following four components: Hb level >18.5 g/dl in men and >16.5 g/dl in women or red cell mass that is >25% above mean normal predicted, or Hb level >17 g/dl in men (>15 g/dl in women) associated with a sustained increase of ⩾2 g/dl from baseline that cannot be attributed to correction of iron deficiency. Altogether it has been argued that application of these criteria may result in an underdiagnosis of PV by excluding patients with actual red cell mass that is 25% above mean predicted value, but whose Hb and Hct levels are below the WHO guidelines.^49, 50, 51, 52, 53, 54, 55, 56^ Recently, the term masked PV (mPV) was re-introduced^57^ for *JAK2*-mutated patients with latent (initial, occult pre-polycythemic) disease manifestations who present with a BM morphology consistent with PV and display persistently raised Hb levels between 16.0 and 18.5 g/dl for men and 15.0 and 16.5 g/dl for women. Subsequently, a Hb level of 16.5 g/dl in men and 16.0 g/dl for women or in accordance with the BCSH criteria,^58^ a Hct level of 49% in men and 48% in women was determined to be the optimal cutoff levels for distinguishing *JAK2*-mutated ET from mPV.^59, 60^ These data were entered in the new proposal for the WHO^28^ and accordingly shown in Table 1. The clinical relevance of recognizing mPV was demonstrated in a recent study on 66 *JAK2*-mutated patients younger than 40 years who revealed a higher risk of thrombosis compared with a control group of 97 cases with overt PV due to the less frequent use of phlebotomies or cytoreductive treatment.^61^ Altogether based on these clinical findings and the fact that BM morphology is superimposable to that of overt PV,^62^ according to the original 2008 criteria,^3, 4^ it makes little sense that the term mPV is maintained, because it may be argued that this entity is in fact fully consistent with PV. Finally, the use of the endogenous erythroid colony growth assay was discarded as a minor diagnostic criterion^28^ due to limited practicability (time consuming, no standardization, restricted to specialized institutions, costly).

Concerning BM morphology in PV there is still some discussion regarding its diagnostic validity and particularly the impact in discriminating PV from other MPN entities.^16, 50, 55^ Contrasting this criticism unique characteristics pointing to both early and overt manifestations of disease have been demonstrated by several groups.^56, 57, 63, 64, 65, 66^ Histopathology of the BM includes usually an age-adjusted hypercellularity^67^ due to tri-lineage proliferation (panmyelosis) including erythro–granulo–and megakaryopoiesis. In former studies by the Polycythemia Vera Study Group (PVSG) wide ranges (35–100%) of hematopoietic cellularity were described,^68^ however, without regarding explicitly age-matched reference values.^67^ Very important is that in PV megakaryopoiesis presents without significant morphological abnormalities but conspicuous differences in size (pleomorphy). In a recent study, differentiation between PV and ET seems to pose significant problems revealing a low sensitivity of histological diagnosis.^16^ A surprisingly high frequency of unclassifiable MPN (23–28% compared with 10–15% or even less in other studies),^5, 66, 69^ an incomplete description of megakaryocyte nuclei atypia (failure to include clumsy/cloud-like abnormalities as key feature of atypia),^70, 71, 72, 73^ but particular a very low reproducibility of fiber grading was in sharp contrast with previously reported findings.^74^ In regard to BM fibers, a minor increase in reticulin fibrosis at diagnosis of PV was reported in less than 20% of biopsy specimens by several groups,^75, 76, 77, 78^ probably related to duration of the disease process preceding diagnostic BM biopsy examination. Concerning the PVSG cohort of PV patients 25% showed a slight and 11% a moderate to marked increase in reticulin.^68, 79^ An increase in reticulin in PV has been associated with worse clinical outcome associated with a more rapid progression to post-PV myelofibrosis.^78^ For this reason, a BM biopsy may even be recommended in cases presenting with sustained absolute erythrocytosis (Hb levels>18.5 g/dl in men—Hct 55.5% or >16.5% g/dl in women—Hct 49.5%) together with all the other criteria (Table 1). Finally, several groups of hematopathologists have validated the specific histological BM pattern characterizing PV^56, 57, 62, 63, 64, 65, 66^ and confirmed and extended the notion that there are no significant differences regarding BM morphology between mPV and overt stages of PV. Based on data, BM morphology as essentially defined by the WHO classification^3, 4^ was promoted to a major criterion in the proposed set of diagnostic criteria.

## Essential thrombocythemia

Concerning ET the obvious need to lower the diagnostic threshold level for the platelet value originally introduced by the PVSG^80^ has been recognized not only by the 2007/2008 updated WHO classification,^3, 4^ but also by the BCSH (Table 2).^13, 14^ Ample evidence has been produced that the arbitrarily chosen limit for a sustained platelet count of 600 × 10^9^/l by the PVSG^80^ fails to consider patients with initial ET associated with a lower degree of thrombocythemia, who may occasionally present with relevant thromboembolic or hemorrhagic complications.^2, 81, 82, 83^ Moreover, many investigators have effectively argued that the use of such a high threshold value is not consistent with the 95th percentile for the normal platelet count adjusted for gender and race being below 400 × 10^9^/l.^2, 84^ For this reason, a cutoff level of 450 × 10^9^/l was proposed in the diagnostic guidelines by the WHO and BCSH^2, 3, 13, 14^ in particular when associated with clinical signs and symptoms that are usually not encountered in reactive thrombocytosis.^85, 86^ Concerning the other hematological parameters in accordance with the BM features (Table 2) in more than 1150 patients with strictly WHO-defined ET collected in three centers, leuko- or erythrocytosis was an unusual finding as well as the occurrence of palpaple splenomegaly or increased levels of lactate dehydrogenase (LDH) while leukoerythroblastosis or poikilocytosis was absent.^5, 6^

In comparison with the 2007/2008 updated WHO criteria,^3, 4^ in ET the BM features have not to be changed significantly by the new proposals but there is the need to define more clearly the differentiation from prePMF. In this regard, the predominance of large to giant, mature megakaryocytes with hyperlobulated nuclei and a random distribution or loose clustering within the BM space without significant atypia is emphasized (Table 2). According to our experience and that of others it seems to be necessary to highlight explicitly that there is usually no or very rarely, less than 5%, a minor-grade 1^(ref. 67)^ increase in reticulin fibers in WHO-defined ET at onset^5, 6, 70, 75, 76, 77^ to avoid any confusion with PMF as has been reported.^87, 88, 89^ This is an important finding to reduce possible shortcomings attributed to subjectivity in interpreting BM morphology, standardization of prominent BM parameters is a key issue^70, 90^ and an essential feature, especially in PV cases presenting clinically with an ET-like appearance.^63, 64, 65^ The attempted aim is to generate a histological pattern characterizing the different subtypes of MPN.^71^ Overall application of the WHO-defined BM criteria on larger cohorts of patients either blindly or as explicitly postulated, in context with clinical data has resulted in consensus rates ranging between 73% and more than 90% largely depending on study design (all subtypes of MPN, inclusion of control cases with reactive changes, restriction to single BM parameters or only ET versus PMF, blinded evaluation or consideration of clinical data).^5, 6, 66, 69, 72, 73^ On the other hand, several groups^16, 87, 88, 91, 92^ failed to reproduce the WHO diagnostic guidelines to a sufficient extend for a variety of reasons that have been reviewed and detailed^15^ or previously discussed in context with PV.^16^ Among others these included, failing standardization of BM features, inability of morphological pattern recognition, accurate fiber grading or small biopsy specimens. In this context, it has to be conceded that up to 10–15% of patients may present with MPN unclassifiable.^5, 66, 69^ In practice, this rate depends significantly on the experience of the reviewer, the presence of very prodromal or terminal manifestations of disease, the lack of information about cytoreductive treatment and incomplete clinical data and mutation status.

## Primary myelofibrosis

Overt/advanced stages of PMF are clinically characterized by marked anemia, borderline to mild leukocytosis or leukopenia, slight thrombocytosis or even decrease in platelet counts, high levels of serum LDH, the presence of peripheral erythro and myeloblasts (leukoerythroblastosis), tear drop-shaped erythrocytes and gross splenomegaly (that is, myelofibrosis with myeloid metaplasia).^11, 93^ All these features (Table 3) are not fully or only borderline expressed in prePMF (Table 4).^7, 94, 95^ Disease evolution can be easily observed in cohorts of patients followed over many years with sequential BM biopsy examinations^96, 97^ thus elucidating the stepwise progression of PMF initiating from a prodromal (prefibrotic) stage without an increase in BM reticulin.^7^ Although the existence of a prodromal stage of PMF, that is, prePMF is supported by several studies, its clear-cut differentiation from ET and the diagnostic impact of clinical parameters have been questioned.^13, 89, 91^ Previous reports by a number of authorities are in full agreement that by strictly regarding the corresponding WHO criteria,^2, 3, 4^ an accurate discrimination between ET and prePMF can be accomplished that exerts a significant impact on presentation and clinical outcome.^5, 6, 7, 9, 66, 94, 95, 98, 99^ Altogether significant differences of presenting clinical data were observed between prePMF and ET regarding leukocyte counts (prePMF>ET), Hb level (prePMF<ET), LDH values (prePMF>ET), occurrence of a few peripheral erythro- or myeloblasts (prePMF>ET) and finally, incidence of palpable splenomegaly (prePMF>ET).^5, 6^ In aggregate, in association with characteristic BM morphology, these clinical features allow an accurate distinction between prePMF and ET in the majority of cases provided in multicenter studies.^5, 6, 66, 98^

The key features of prePMF usually include increased age-matched BM cellularity, increased megakaryopoiesis of small to large megakaryocytes with atypical histotopography (endosteal translocation, dense clusters), and distinctive nuclear features (hypolobulation, clumsy-clould-like, maturation defects) and granulocytic proliferation, whereas erythropoiesis is usually reduced (Table 4). There is either a normal or only minor, amount of reticulin fibers present (grades 0/1) contrasting advanced reticulin/collagen fibrosis (grades 2/3) in overt PMF.^67^ According to several investigations by different groups, largely depended on methods and study design, consensus among diagnosis in distinguishing ET from prePMF diagnoses ranged from 53 to 88%.^5, 6, 9, 66, 69, 72, 73^ Contradicting the agreement on morphological features the current ‘minor clinical criteria' for the diagnosis of prePMF^3, 4^ sometimes impaired accurate classification and a clear-cut separation of prePMF from ET.^91^

Finally, concerning the still controversial differentiation between ET and prePMF^13, 14, 15, 73, 88, 90, 91, 92^ experts discuss that a more easy and precise definition of prePMF may be obtained by moving from a qualitative morphological description and a pattern recognition diagnostic process to a more quantitative evaluation of the morphological hallmarks and to follow a standardized procedural diagnostic pathway.^71, 90^ Although this is a very convincing proposal that has to be regarded, considering the overall poor results of fiber grading by local pathologists^100^ in an international multicenter evaluation study, we should be aware that until now only few reference centers would be able to recognize and apply these key diagnostic features. All these shortcomings call for launching workshops and training sessions involving hematopathologists in reference centers to improve this unwanted situation we are still facing today. It is evident that this adverse situation may add to the conflict of opinion occasionally expressed concerning the overall value of BM morphology in MPN diagnosis.

## Conclusion

Following the WHO classification the golden yardstick to accomplish a final diagnosis consists always of a synoptical approach including clinical and morphological findings and whenever possible, disease-relevant mutations. Keeping these postulates in mind the 2007/2008 updated WHO diagnostic criteria provided already a very solid basis now amenable to further improvement after more than 6 years of practical experience. The proposed amendments regard not only newly discovered molecular markers, but also the introduction of so-called mPV including a lowering of Hb and Hct values and an upgrading of BM morphology as major criterion. Moreover, there is a need for a more precise description of histopathology focused on fibrosis in ET and of minor clinical features in prePMF. For the future biological markers may probably be expected to be more useful for the discrimination of the different MPN subtypes in accordance with the histological BM pattern and corresponding clinical features.

## Figures and Tables

**Table 1:** Evolution of the WHO diagnostic guidelines for MPN between 2001 and 2015

	*WHO criteria 2001*	*WHO criteria 2008*	*Proposed WHO criteria 2015 (adapted with minor modifications)*
*Polycythemia vera (PV)*			
Hemoglobin, g/dl Red cell mass Hematocrit, %	A1 ->18.5 in men >16.5 in women or increased	A1 ->18.5 in men >16.5 in women or increased increased	A1 ->16.5 in men >16.0 in women or increased >49% in men >48% in women
Secondary erythrocytosis	A2 - no evidence		
Splenomegaly	A3 - yes/no		
Clonal genetic abnormality	A4 - yes/no	A2 - JAK2 V617F or JAK2 exon 12 mutation	A3 - JAK2 mutation
Endogenous erythroid colony formation		B3 - yes/no	
Thrombocytosis >400 × 10^9^/l	A5 - yes/no		
Subnormal serum erythropoietin level		B2 - yes/no	B - yes/no
White blood cell counts>12 × 10^9^/l	B2 - yes/no		
Bone marrow (BM) histology	B3 - BM with panmyelosis showing prominent erythroid and megakaryocytic proliferation	B1 - BM with age-adjusted hypercellularity and trilineage growth (panmyelosis)	A2 - BM with age-adjusted hypercellularity and trilineage growth (panmyelosis) with pleomorphic, mature megakaryocytes
Diagnosis of PV requires	A1+A2 and any other category A or A1+A2 and any two of category B	A1+A2 and one B criterion or A1+two B criteria	A1–3 or A1+2 and the B criterion

Abbreviations: MPN, myeloproliferative neoplasm; WHO, World Health Organization. A category: major criteria; B category: minor criteria.

**Table 2:** Evolution of the WHO diagnostic guidelines for MPN between 2001 and 2015

	*WHO criteria 2001*	*WHO criteria 2008*	*Proposed WHO criteria 2015 (adapted with minor modifications)*
*Essential thrombocythemia (ET)*			
Platelet count	A1 -⩾600 × 10^9^/l	A1 -⩾450 × 10^9^/l	A1 -⩾450 × 10^9^/l
Bone marrow (BM) histology	A2 - BM biopsy showing proliferation mainly of the megakaryocytic lineage with increased numbers of enlarged, mature megakaryocytes	A 2 - BM biopsy showing proliferation mainly of the megakaryocytic lineage with increased numbers of enlarged, mature megakaryocytes. No significant increase or left-shift of neutrophil granulopoiesis or erythropoiesis	A2 - BM biopsy showing proliferation mainly of the megakaryocytic lineage with increased numbers of enlarged, mature megakaryocytes. No significant increase or left-shift of neutrophil granulopoiesis or erythropoiesis and very rarely minor (grade 1) increase in reticulin fibers
Criteria of exclusion	A3 - No evidence of WHO-defined PV, CML, PMF, MDS or reactive thrombocytosis	A3 - Not meeting WHO criteria for *BCR-ABL+*CML, PV, PMF, MDS or other myeloid neoplasm	A3 - Not meeting WHO criteria for *BCR-ABL+*CML, PV, PMF, MDS or other myeloid neoplasm
Clonal genetic abnormality		A4 *- JAK2 V617F* or other clonal marker or in the absence, no evidence for reactive thrombocytosis	A4 - Presence of *JAK2, CALR* or *MPL* mutation
Minor criteria			B - Presence of a clonal marker or absence of evidence for reactive thrombocytosis
Diagnosis of ET requires	A1–A4	A1–A 4	A1–A4 or A1–A3 and one of the B criteria

Abbreviations: CML, chronic myeloid leukemia; ET, essential thrombocythemia; MDS, myelodysplastic syndromes; MPN, myeloproliferative neoplasm; PMF, primary myelofibrosis; PV, polycythemia vera; WHO, World Health Organization.

A category: major criteria; B category: minor criteria.

**Table 3:** Evolution of the WHO diagnostic guidelines for MPN between 2001 and 2015

	*WHO criteria 2001*	*WHO criteria 2008*	*Proposed WHO criteria 2015 (adapted with minor modifications)*
*Prefibrotic/early primary myelofibrosis (prePMF)*			
Bone marrow (BM) histology	BM biopsy with hypercellularity, neutrophilic proliferation and megakaryocytic proliferation and atypia (clustering, abnormally lobulated nuclei, naked nuclei). Minimal or absent reticulin fibrosis	A1 - BM biopsy showing megakaryocytic proliferation and atypia, in the absence of significant reticulin fibrosis, megakaryocytic changes must be accompanied by increased cellularity, granulocytic proliferation and often decreased erythropoiesis	A1 - BM biopsy showing megakaryocytic proliferation and atypia without reticulin fibrosis >grade 1, accompanied by increased age-adjusted cellularity, granulocytic proliferation and often decreased erythropoiesis
Criteria of exclusion		A2 - Not meeting WHO criteria for *BCR-ABL+*CML, MDS or other myeloid neoplasm	A2 - Not meeting WHO criteria for *BCR-ABL+*CML, PV, ET, MDS or other myeloid neoplasm
Clonal genetic abnormality		A3 - *JAK2 V617F* or other clonal marker (*MPL*) or in the absence, no evidence for reactive fibrosis^a^	A3 - Presence of *JAK2, CALR* or *MPL mutation* or in the absence, presence of an other clonal marker^b^ or no evidence for reactive reticulin fibrosis
Clinical findings	Mild anemia, mild to moderate leukocytosis, mild to marked thrombocytosis, no or mild spleno- or hepatomegaly, no or mild leukoerythroblastosis or red blood cell poikilocytosis, few if any dacryocytes	B - criteria (1) Leukoerythroblastosis (2) LDH increase (3) Anemia (4) Splenomegaly	B - criteria (1) Anemia (2) Leukocytosis >11 K/μl (3) Palpable splenomegaly (4) LDH increase
Diagnosis of prePMF requires	All criteria without further specification	A1 –A3 and two of the B criteria	A1-A3 and at least one of the B criteria

Abbreviations: CML, chronic myeloid leukemia; ET, essential thrombocythemia; LDH, serum lactate dehydrogenase increased to above upper normal limit of institutional reference range; MDS, myelodysplastic syndromes; MPN, myeloproliferative neoplasm; PV, polycythemia vera; WHO, World Health Organization.

A category: major criteria; B category: minor criteria.

a In the absence of any of the three major clonal mutations, the search for the most frequent accompanying mutations *(ASXL1, EZH2, TET2, IDH1/IDH2, SRSF2, SF3B1)* is of help in determining the clonal nature of the disease.

b Minor bone marrow reticulin fibrosis secondary to infection, autoimmune disorder or other chronic inflammatory conditions, hairy cell leukemia or other lymphoid neoplasm, metastatic malignancy or toxic (chronic) myelopathies.

**Table 4:** Evolution of the WHO diagnostic guidelines for MPN between 2001 and 2015

	*WHO criteria 2001*	*WHO criteria 2008*	*Proposed WHO criteria 2015 (adapted with minor modifications)*
*Primary myelofibrosis (PMF)*			
Bone marrow (BM) histology	BM biopsy with decreased cellularity, dilated marrow sinuses with intraluminal hematopoiesis, prominent megakaryocytic proliferation and atypia (clustering, abnormally lobulated nuclei, naked nuclei), reticulin and/or collagen fibrosis, new bone formation (osteosclerosis)	A1 - BM biopsy showing megakaryocytic proliferation and atypia, usually accompanied by either reticulin or collagen fibrosis	A1 - BM biopsy showing megakaryocytic proliferation and atypia accompanied by either reticulin or collagen fibrosis grades 2 or 3
Criteria of exclusion		A2 - Not meeting WHO criteria for *BCR-ABL+*CML, MDS or other myeloid neoplasm	A2 - Not meeting WHO criteria for *BCR-ABL+*CML, PV, ET, MDS or other myeloid neoplasm
Clonal genetic abnormality		A3 - *JAK2 V617F* or other clonal marker (*MPL*) or in the absence, no evidence for reactive fibrosis^a^	A3 - Presence of *JAK2, CALR* or *MPL* mutation or in the absence, presence of another clonal marker^b^ or no evidence for reactive reticulin fibrosis^a^
Clinical findings	Moderate to marked anemia,low,normal or elevated WBC, platelet count low,normal or elevated, moderate to marked spleno-or hepatomegaly, leukoerythroblastosis, prominent red blood cell poikilocytosis with dacryocytes	B - criteria (1) Leukoerythroblastosis (2) LDH increase (3) Anemia (4) Splenomegaly	B - criteria (1) Anemia (2) Leukocytosis >11 K/μl (3) Palpable splenomegaly (4) LDH increase (5) Leukoerythroblastosis
Diagnosis of prePMF requires	All criteria without further specification	A1–A3 and two of the B criteria	A1–A3 and at least one of the B criteria

Abbreviations: CML, chronic myeloid leukemia; ET, essential thrombocythemia; LDH, serum lactate dehydrogenase increased to above upper normal limit of institutional reference range; MDS, myelodysplastic syndromes; MPN, myeloproliferative neoplasm; PV, polycythemia vera; WBC, white blood cell count; WHO, World Health Organization.

A category: major criteria; B category: minor criteria.

a In the absence of any of the three major clonal mutations, the search for the most frequent accompanying mutations (*ASXL1, EZH2, TET2, IDH1/IDH2, SRSF2, SF3B1*) is of help in determining the clonal nature of the disease.

b Bone marrow fibrosis secondary to infection, autoimmune disorder or other chronic inflammatory conditions, hairy cell leukemia or other lymphoid neoplasm, metastatic malignancy or toxic (chronic) myelopathies.
